# Supplementary material for: scDSSC: Deep Sparse Subspace Clustering for scRNA-seq Data
Source: PLoS Comput Biol. 2022 Dec 19;18(12):e1010772. doi: 10.1371/journal.pcbi.1010772 (PMC9810169; doi:10.1371/journal.pcbi.1010772)
Supplement: S1 Note — Here we describe ACC, NMI, and ARI in detail, and give their calculation process. (DOCX) [file pcbi.1010772.s001.docx]

**scDSSC: Deep Sparse Subspace Clustering for scRNA-seq Data**

Running head: Deep Sparse Subspace Clustering

HaiYun Wang ^1^, Jianping Zhao ^1,^ *, ChunHou Zheng ^2^, YanSen Su^2,^ *

1 College of Mathematics and System Sciences, Xinjiang University, Urumqi, China, 2 School of Artifial Intelligence, Anhui University, Hefei, China

***** **zhaojianping@126.com (Z-JP); suyansen1985@163.com (S-YS)**

# Supplementary Materials

### Note S1 Evaluation Metrics

In this paper, Clustering Accuracy (CA) [1], Normalized Mutual Information (NMI) [2] and Adjusted Rand Index (ARI) [3] are used to compare the performance of different methods.

Let $U=\left\{ U_{1},U_{2},\cdots U_{C_{U}} \right\}$ and $V=\left\{ V_{1},V_{2},\cdots V_{C_{V}} \right\}$ be the predicted and ground-truth clusters on a set of n data points. NMI is formally defined as:

$NMI=\frac{I(U,V)}{max\{H\left( U \right),H(V)\}},$ (1)

where $I\left( U,V \right)=\sum_{p=1}^{C_{U}} \sum_{q=1}^{C_{V}} |U_{p}\cap V_{q}|log\frac{n|U_{p}\cap V_{q}|}{|U_{p}|\times|V_{q}|}$, which means the mutual information between $U$ and $V$; $H\left( U \right)=-\sum_{p=1}^{C_{U}} |U_{p}|log\frac{|U_{p}|}{n}$ and $H\left( V \right)=-\sum_{q=1}^{C_{V}} |V_{q}|log\frac{|V_{q}|}{n}$ are the entropies.

CA represents the best matching between the ground-truth clusters and predicted clusters. The CA is defined as follows:

$CA=\max_{m} \sum_{i=1}^{n} 1\frac{\{l_{i}=m(\hat{l}_{i})\}}{n},$ (2)

where $l_{i}$ and $\hat{l}_{i}$ are the ground-truth label and predicted label respectively for data point i, n refers to the number of data points, m ranges over all possible one-to-one mapping between predicted labels and true labels.

The Rand Index is used to evaluate the similarity between cluster assignment U and V in data clustering. However, the corrected-for-chance version of the former Adjust Rand Index (ARI) will be used more often. The ARI is calculated as follows:

$ARI=\frac{\binom{n}{2}\left( a+d \right)-[\left( a+b \right)\left( a+c \right)+(c+d)(b+d)]}{\binom{n}{2}-[\left( a+b \right)\left( a+c \right)+(c+d)(b+d)]}$. (3)

Where *a* is the number of pairs of two objects in the same group in both *U* and *V*; *b* is the number of pairs of two objects in different groups in both *U* and *V*; *c* is the number of pairs of two objects in the same group in *U* but in different groups in *V*; and *d* is the number of pairs of two objects in different groups in *U* but in the same group in *V*.

# References

1. Xie, J., Girshick, R., & Farhadi, A, “Unsupervised deep embedding for clustering analysis, in International conference on machine learning (PMLR), 2016, pp. 478-487.
2. Strehl, A., & Ghosh, J, “Cluster ensembles---a knowledge reuse framework for combining multiple partitions,” Journal of machine learning research, vol. 3, pp. 583-617, Dec. 2003.
3. Qi, R., Ma, A., Ma, Q., & Zou, Q, “Clustering and classification methods for single-cell RNA-sequencing data,” Briefings in bioinformatics, vol. 21, no. 4, pp. 1196-1208, Jul. 2020.
