# Supplementary figures and images for: scDSSC: Deep Sparse Subspace Clustering for scRNA-seq Data
Source: PLoS Comput Biol. 2022 Dec 19;18(12):e1010772. doi: 10.1371/journal.pcbi.1010772 (PMC9810169; doi:10.1371/journal.pcbi.1010772)

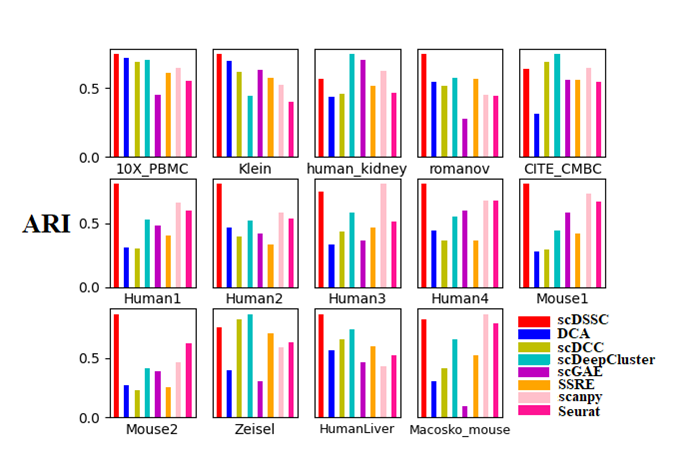

Supplement: S1 Fig — Each subgraph represents the clustering results of eight clustering methods on a dataset. Different colors correspond to different methods, and the ordinate represents the ARI score. (TIF) [file pcbi.1010772.s002.tif]

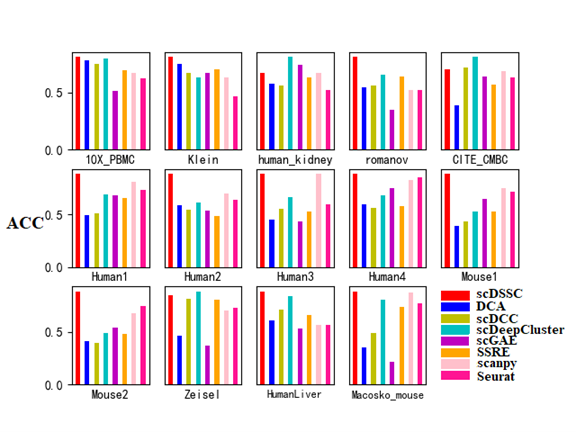

Supplement: S2 Fig — (TIF) [file pcbi.1010772.s003.tif]

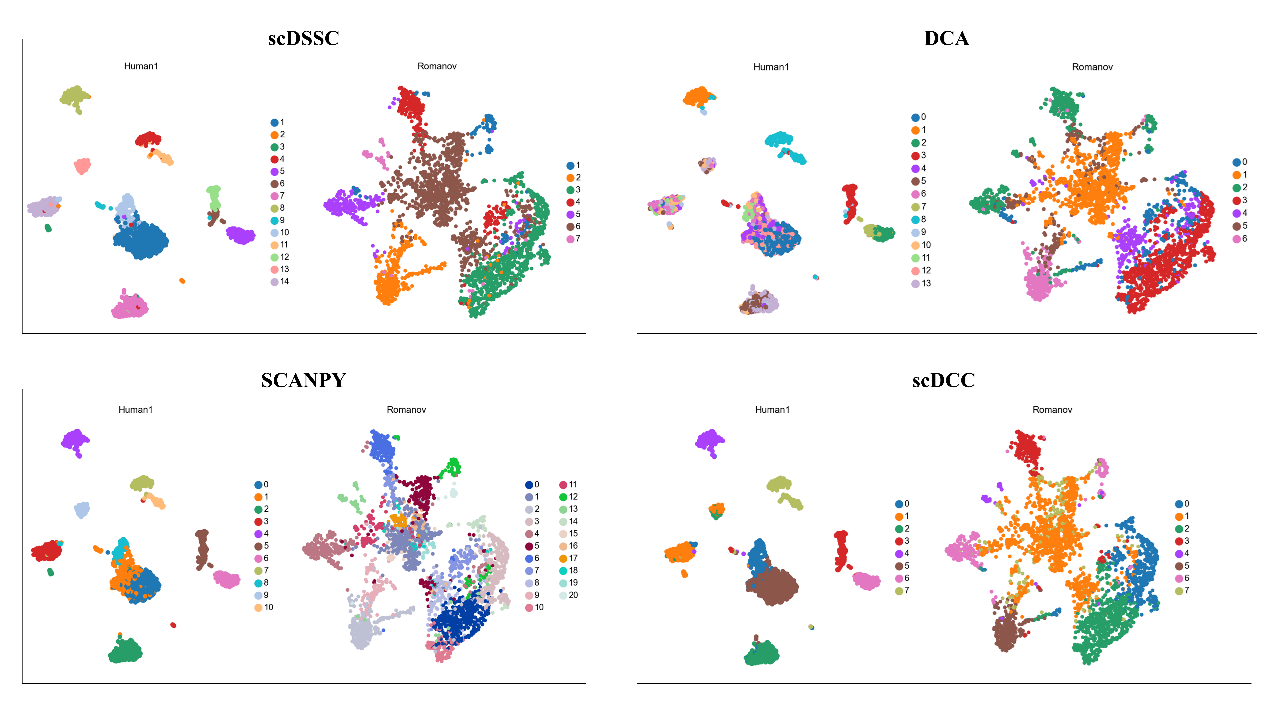

Supplement: S3 Fig — Here, we show the visualization results of scDSSC, DCA, SCANPY and scDCC respectively. The results corresponding to each method are composed of two subgraphs. The left figure represents the visualization results on Human1 dataset, and the right figure represents the visualization results on Romaov dataset. (TIF) [file pcbi.1010772.s004.tif]

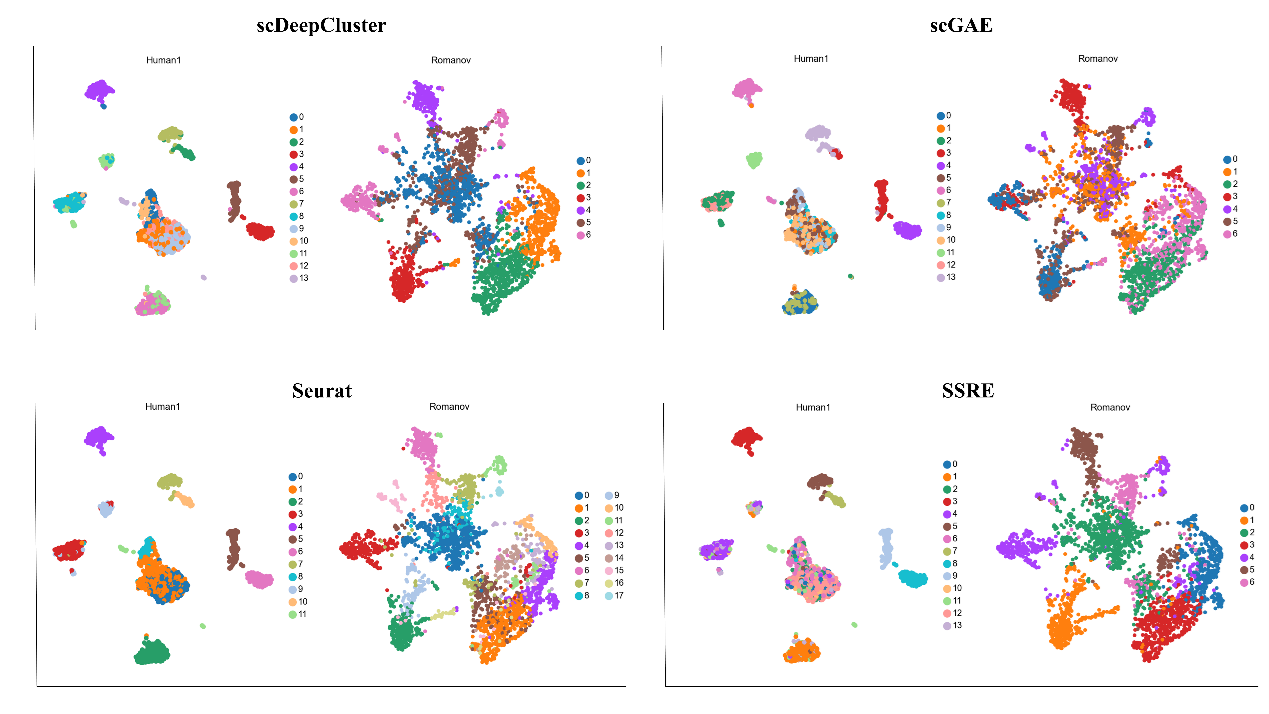

Supplement: S4 Fig — Here, we show the visualization results of scDeepCluster, scGAE, Seurat and SSRE respectively. (TIF) [file pcbi.1010772.s005.tif]

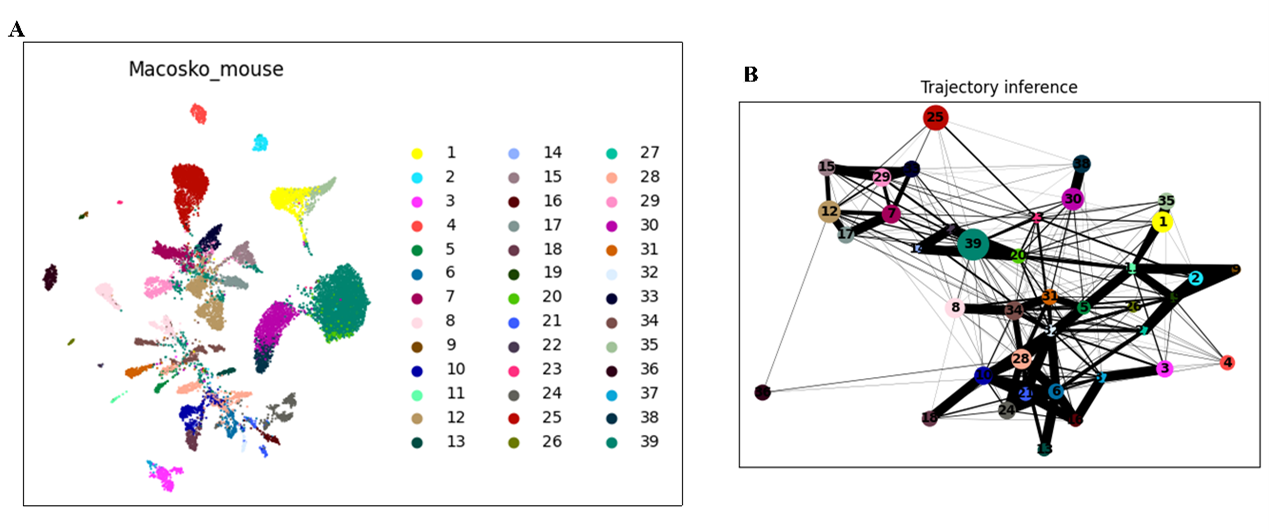

Supplement: S5 Fig — Plot A shows the cell visualization results, and plot B shows the trajectory inference results. (TIF) [file pcbi.1010772.s006.tif]
