# Supplementary material for: scDSSC: Deep Sparse Subspace Clustering for scRNA-seq Data
Source: PLoS Comput Biol. 2022 Dec 19;18(12):e1010772. doi: 10.1371/journal.pcbi.1010772 (PMC9810169; doi:10.1371/journal.pcbi.1010772)
Supplement: S1 Table — We downloaded the Macosko dataset and filtered cells and genes. Concretely, cells with <700 genes and genes with <3 reads in 3 cells were filtered out. As a result, we obtained 14,653 cells by 11,422 genes among 39 clusters. For CITE_CMBC dataset, we selected the top 2000 dispersed genes to conduct clustering experiments. And for HumanLiver datset, we selected the top 5000 dispersed genes to conduct clustering experiments. (DOCX) [file pcbi.1010772.s007.docx]

**S1 Table** The details of datasets used in this paper. We downloaded the Macosko dataset and filtered cells and genes. Concretely, cells with <700 genes and genes with <3 reads in 3 cells were filtered out. As a result, we obtained 14,653 cells by 11,422 genes among 39 clusters. For CITE_CMBC dataset, we selected the top 2000 dispersed genes to conduct clustering experiments. And for HumanLiver datset, we selected the top 5000 dispersed genes to conduct clustering experiments.

| Dataset | Cell number | Gene number | Cell types | Source |
| --- | --- | --- | --- | --- |
| 10X_PBMC | 4271 | 16653 | 8 | [1] |
| Klein | 2717 | 24175 | 4 | [2] |
| Human_kidney | 5685 | 25215 | 11 | [3] |
| CITE_CMBC | 8617 | 2000 | 15 | [4] |
| Romanov | 2881 | 24341 | 7 | [5] |
| Human1 | 1937 | 20125 | 14 | [6] |
| Human2 | 1724 | 20125 | 14 | [6] |
| Human3 | 3605 | 20125 | 14 | [6] |
| Human4 | 1303 | 20125 | 14 | [6] |
| Mouse1 | 822 | 14878 | 13 | [6] |
| Mouse2 | 1064 | 14878 | 13 | [6] |
| Zeisel | 3005 | 19972 | 9 | [7] |
| HumanLiver | 8444 | 5000 | 11 | [8] |
| Macosko_mouse | 14653 | 11422 | 39 | [9] |

**References**

1. Zheng, G. X., Terry, J. M., Belgrader, P., Ryvkin, P., Bent, Z. W., Wilson, R., ... & Bielas, J. H. (2017). Massively parallel digital transcriptional profiling of single cells. Nature communications, 8(1), 1-12.
2. Klein, A. M., Mazutis, L., Akartuna, I., Tallapragada, N., Veres, A., Li, V., ... & Kirschner, M. W. (2015). Droplet barcoding for single-cell transcriptomics applied to embryonic stem cells. Cell, 161(5), 1187-1201.
3. Young, M. D., Mitchell, T. J., Vieira Braga, F. A., Tran, M. G., Stewart, B. J., Ferdinand, J. R., ... & Behjati, S. (2018). Single-cell transcriptomes from human kidneys reveal the cellular identity of renal tumors. science, 361(6402), 594-599.
4. Mimitou, E. P., Cheng, A., Montalbano, A., Hao, S., Stoeckius, M., Legut, M., ... & Smibert, P. (2019). Multiplexed detection of proteins, transcriptomes, clonotypes and CRISPR perturbations in single cells. Nature methods, 16(5), 409-412.
5. Romanov, R. A., Zeisel, A., Bakker, J., Girach, F., Hellysaz, A., Tomer, R., ... & Harkany, T. (2017). Molecular interrogation of hypothalamic organization reveals distinct dopamine neuronal subtypes. Nature neuroscience, 20(2), 176-188.
6. Baron, M., Veres, A., Wolock, S. L., Faust, A. L., Gaujoux, R., Vetere, A., ... & Yanai, I. (2016). A single-cell transcriptomic map of the human and mouse pancreas reveals inter-and intra-cell population structure. Cell systems, 3(4), 346-360.
7. Zeisel, A., Muñoz-Manchado, A. B., Codeluppi, S., Lönnerberg, P., La Manno, G., Juréus, A., ... & Linnarsson, S. (2015). Cell types in the mouse cortex and hippocampus revealed by single-cell RNA-seq. Science, 347(6226), 1138-1142.
8. Tian, T., Zhang, J., Lin, X., Wei, Z., & Hakonarson, H. (2021). Model-based deep embedding for constrained clustering analysis of single cell RNA-seq data. Nature communications, 12(1), 1-12.
9. Macosko, E. Z., Basu, A., Satija, R., Nemesh, J., Shekhar, K., Goldman, M., ... & McCarroll, S. A. (2015). Highly parallel genome-wide expression profiling of individual cells using nanoliter droplets. Cell, 161(5), 1202-1214.
