# Supplementary material for: scDSSC: Deep Sparse Subspace Clustering for scRNA-seq Data
Source: PLoS Comput Biol. 2022 Dec 19;18(12):e1010772. doi: 10.1371/journal.pcbi.1010772 (PMC9810169; doi:10.1371/journal.pcbi.1010772)
Supplement: S2 Table — The results here correspond to S1 Fig. Here are the ARI scores of eight methods on each dataset. (DOCX) [file pcbi.1010772.s008.docx]

S2 Table The clustering performance accessed by ARI. The results here correspond to S1 Fig. Here are the ARI scores of eight methods on each dataset.

| Dataset | scDSSC | DCA | scDCC | scDeepCluster | scGAE | SSRE | scanpy | Seurat |
| --- | --- | --- | --- | --- | --- | --- | --- | --- |
| 10X_PBMC | 0.7476 | 0.7201 | 0.6903 | 0.7055 | 0.4492 | 0.6099 | 0.6414 | 0.5513 |
| Klein | 0.9161 | 0.8496 | 0.7592 | 0.5426 | 0.7759 | 0.7045 | 0.6408 | 0.4891 |
| Human_kidney | 0.5152 | 0.4001 | 0.4156 | 0.6845 | 0.6404 | 0.4732 | 0.5738 | 0.4223 |
| CITE_CMBC | 0.5816 | 0.2836 | 0.6317 | 0.6828 | 0.5105 | 0.5085 | 0.5914 | 0.4988 |
| romanov | 0.6799 | 0.4925 | 0.4681 | 0.5201 | 0.2512 | 0.5161 | 0.4060 | 0.4043 |
| Human1 | 0.8086 | 0.3132 | 0.3059 | 0.5293 | 0.4840 | 0.4047 | 0.6602 | 0.6012 |
| Human2 | 0.8855 | 0.5072 | 0.4363 | 0.5699 | 0.4605 | 0.3698 | 0.6393 | 0.5921 |
| Human3 | 0.8082 | 0.3667 | 0.4768 | 0.6361 | 0.3933 | 0.5034 | 0.8782 | 0.5542 |
| Human4 | 0.8335 | 0.4581 | 0.3773 | 0.5678 | 0.6181 | 0.3775 | 0.6999 | 0.6990 |
| Mouse1 | 0.7258 | 0.2514 | 0.2662 | 0.3980 | 0.5261 | 0.3770 | 0.6566 | 0.6018 |
| Mouse2 | 0.8678 | 0.2725 | 0.2249 | 0.4141 | 0.3845 | 0.2539 | 0.4658 | 0.6242 |
| Zeisel | 0.6391 | 0.3383 | 0.7008 | 0.7354 | 0.2550 | 0.5969 | 0.4953 | 0.5375 |
| HumanLiver | 0.8447 | 0.5490 | 0.6405 | 0.7169 | 0.4515 | 0.5781 | 0.4148 | 0.5052 |
| Macosko_mouse | 0.8010 | 0.2985 | 0.3986 | 0.6352 | 0.0925 | 0.5045 | 0.8427 | 0.7702 |
