# Supplementary material for: scDSSC: Deep Sparse Subspace Clustering for scRNA-seq Data
Source: PLoS Comput Biol. 2022 Dec 19;18(12):e1010772. doi: 10.1371/journal.pcbi.1010772 (PMC9810169; doi:10.1371/journal.pcbi.1010772)
Supplement: S3 Table — The results here correspond to S2 Fig. Here are the NMI scores of eight methods on each dataset. (DOCX) [file pcbi.1010772.s009.docx]

**S3 Table** The clustering performance accessed by NMI. The results here correspond to S2 Fig. Here are the NMI scores of eight methods on each dataset.

| Dataset | scDSSC | DCA | scDCC | scDeepCluster | scGAE | SSRE | scanpy | Seurat |
| --- | --- | --- | --- | --- | --- | --- | --- | --- |
| 10X_PBMC | 0.7592 | 0.7335 | 0.7291 | 0.7706 | 0.6600 | 0.6726 | 0.6983 | 0.6714 |
| Klein | 0.8831 | 0.8571 | 0.8216 | 0.6994 | 0.7860 | 0.7190 | 0.6997 | 0.7091 |
| Human_kidney | 0.6582 | 0.5783 | 0.6319 | 0.7712 | 0.7491 | 0.6162 | 0.7360 | 0.6542 |
| CITE_CMBC | 0.6697 | 0.5217 | 0.7227 | 0.7788 | 0.7031 | 0.6747 | 0.7746 | 0.7123 |
| romanov | 0.7068 | 0.5159 | 0.5383 | 0.5388 | 0.5512 | 0.6230 | 0.5992 | 0.6312 |
| Human1 | 0.8707 | 0.5755 | 0.5772 | 0.7929 | 0.7597 | 0.6658 | 0.8386 | 0.8365 |
| Human2 | 0.8590 | 0.7042 | 0.6257 | 0.7586 | 0.7494 | 0.6258 | 0.8315 | 0.7775 |
| Human3 | 0.8009 | 0.5671 | 0.6592 | 0.7891 | 0.6807 | 0.6689 | 0.8915 | 0.7672 |
| Human4 | 0.8268 | 0.6898 | 0.5383 | 0.7592 | 0.7873 | 0.5714 | 0.8362 | 0.8202 |
| Mouse1 | 0.7881 | 0.5227 | 0.5252 | 0.6853 | 0.7204 | 0.6589 | 0.7897 | 0.7918 |
| Mouse2 | 0.8092 | 0.5623 | 0.4499 | 0.6410 | 0.6287 | 0.5510 | 0.7298 | 0.8136 |
| Zeisel | 0.6340 | 0.4937 | 0.7510 | 0.7336 | 0.5664 | 0.6354 | 0.6551 | 0.6732 |
| HumanLiver | 0.8130 | 0.6700 | 0.7677 | 0.8169 | 0.7157 | 0.6886 | 0.7389 | 0.7075 |
| Macosko_mouse | 0.8161 | 0.5095 | 0.6458 | 0.8004 | 0.5081 | 0.7288 | 0.8117 | 0.7666 |
