# Supplementary material for: scDSSC: Deep Sparse Subspace Clustering for scRNA-seq Data
Source: PLoS Comput Biol. 2022 Dec 19;18(12):e1010772. doi: 10.1371/journal.pcbi.1010772 (PMC9810169; doi:10.1371/journal.pcbi.1010772)
Supplement: S4 Table — Here, we give the specific parameter values in the process of pre-training and fine-tuning, as well as the value of subspace dimension of different datasets. (DOCX) [file pcbi.1010772.s010.docx]

**S4 Table** Parameter setting. Here, we give the specific parameter values in the process of pre-training and fine-tuning, as well as the value of subspace dimension of different datasets.

| Dataset | Pre-training epoch | Fine-tuning epoch | Subspace dimension |
| --- | --- | --- | --- |
| 10X_PBMC | 200 | 200 | 7 |
| Klein | 200 | 100 | 10 |
| Human_kidney | 200 | 200 | 8 |
| CITE_CMBC | 200 | 200 | 7 |
| romanov | 200 | 200 | 7 |
| Human1 | 200 | 200 | 11 |
| Human2 | 200 | 200 | 9 |
| Human3 | 200 | 200 | 6 |
| Human4 | 200 | 200 | 10 |
| Mouse1 | 200 | 100 | 7 |
| Mouse2 | 200 | 200 | 9 |
| Zeisel | 200 | 200 | 7 |
| HumanLiver | 200 | 100 | 10 |
| Macosko_mouse | 200 | 100 | 8 |
