# Supplementary material for: scDSSC: Deep Sparse Subspace Clustering for scRNA-seq Data
Source: PLoS Comput Biol. 2022 Dec 19;18(12):e1010772. doi: 10.1371/journal.pcbi.1010772 (PMC9810169; doi:10.1371/journal.pcbi.1010772)
Supplement: S5 Table — Three different conditions were set for the whole experiment, namely, the normal scDSSC model, the loss function without MSE and the loss function without ZINB. The metric NMI was used to assess the clustering performance. (DOCX) [file pcbi.1010772.s011.docx]

**S5 Table** Loss function ablation experiment. Three different conditions were set for the whole experiment, namely, the normal scDSSC model, the loss function without MSE and the loss function without ZINB. The metric NMI was used to assess the clustering performance.

| Datasets | MSE+ZINB | Without ZINB | Without MSE |
| --- | --- | --- | --- |
| 10X_PBMC | 0.7592 | 0.5872 | 0.2983 |
| klein | 0.8831 | 0.6462 | 0.2904 |
| romanov | 0.7068 | 0.5609 | 0.2034 |
| Mouse1 | 0.7881 | 0.5531 | 0.4759 |
| Mouse2 | 0.8092 | 0.5825 | 0.4036 |
