# Supplementary material for: scDSSC: Deep Sparse Subspace Clustering for scRNA-seq Data
Source: PLoS Comput Biol. 2022 Dec 19;18(12):e1010772. doi: 10.1371/journal.pcbi.1010772 (PMC9810169; doi:10.1371/journal.pcbi.1010772)
Supplement: S6 Table — (DOCX) [file pcbi.1010772.s012.docx]

**S6 Table** Comparison of the clustering performance of SC3 and scDSSC on 14 datasets.

|  | SC3 | | scDSSC | |
| --- | --- | --- | --- | --- |
| Datasets | NMI | ARI | NMI | ARI |
| Mouse1 | 0.7192 | 0.4237 | 0.7881 | 0.7258 |
| Mouse2 | 0.6297 | 0.2897 | 0.8092 | 0.8678 |
| 10X_PBMC | 0.7416 | 0.7374 | 0.7592 | 0.7476 |
| Human2 | 0.8150 | 0.6507 | 0.8590 | 0.8855 |
| Romanov | 0.4916 | 0.5182 | 0.7068 | 0.6799 |
| Human1 | 0.7800 | 0.5661 | 0.8707 | 0.8086 |
| Human3 | 0.8265 | 0.7204 | 0.8009 | 0.8082 |
| Human4 | 0.8021 | 0.5964 | 0.8268 | 0.8335 |
| Zeisel | 0.7870 | 0.8497 | 0.6340 | 0.6391 |
| Klein | 0.9980 | 0.9993 | 0.8831 | 0.9161 |
| CITE_CBMC | 0.4183 | 0.1320 | 0.6697 | 0.5816 |
| Human_kidney | 0.5964 | 0.4717 | 0.6582 | 0.5152 |
| HumanLiver | 0.5192 | 0.2550 | 0.8130 | 0.8447 |
| Macosko_mouse | 0.3281 | 0.0228 | 0.8161 | 0.8010 |
