# Supplementary material for: scDSSC: Deep Sparse Subspace Clustering for scRNA-seq Data
Source: PLoS Comput Biol. 2022 Dec 19;18(12):e1010772. doi: 10.1371/journal.pcbi.1010772 (PMC9810169; doi:10.1371/journal.pcbi.1010772)
Supplement: Attachment — Submitted filename: Response letter.docx [file pcbi.1010772.s013.docx]

The Authors’ Response to the Reviewer's Comments on PCOMPBIOL-D-22-01159

**“scDSSC: Deep Sparse Subspace Clustering for scRNA-seq Data”**

Hai-Yun Wang, Jian-Ping Zhao, Chun-Hou Zheng and Yan-Sen Su

We sincerely thank the editor and the reviewers for their constructive comments and suggestions concerning our manuscript entitled “scDSSC: Deep Sparse Subspace Clustering for scRNA-seq Data” (ID: PCOMPBIOL-D-22-01159). We have carefully revised the manuscript according to these constructive comments. The responds to the reviewer’s comments are listed as follows.

**Reviewer reports:**

**Reviewer 1:**

**General:**
This paper proposes a deep sparse subspace clustering method scDSSC combining noise reduction and dimensionality reduction for scRNA-seq data. Experiments on a variety of scRNA-seq datasets from thousands to tens of thousands of cells have shown that scDSSC can significantly improve clustering performance. My concerns can be found below.

1. You should describe the data preprocessing process in more detail, such as size factor calculation, normalization, log-transformation, etc., and it would be clearer to give the formulae for their calculation.

Response: Thank you very much for your comments. We have added more details about the data pre-processing process in the section " Read count data preprocessing and transformation " Line 102, including process descriptions, calculation formulas, etc.

2. In your work, the three hyperparameters λ_1, λ_2 and λ_3 are taken as 0.2, 1.0, 0.5 respectively, is there any special condition for their values? What is the basis for the values you have given them?

Response: Thanks for your comments. The loss function of the network consists of four components, which differ significantly in their numerical magnitude. In order to avoid that the part of the loss with larger values plays a dominant role and hinders the original ability of other losses with smaller values, we add different weighting factors in front of each part of the loss. For example, if the reconstruction loss is large, it corresponds to a weighting coefficient $\lambda_{1}$ set to 0.2, while the self-repressive loss is small, then it has a weighting factor $\lambda_{2}$ set to 1. Of course, the specific values of these three hyperparameters are determined based on the experimental results.

3. Is there any connection between pre-training and fine-tuning in the optimization process? Or what are the differences between them?

Response: Thanks for your comments. Pre-training and fine-tuning are two different stages in the optimization process. The connection between them is that fine-tuning is to use different optimizers to continue training the model after pre-training. The difference between them is that the optimizers used are different, which is reflected in the different learning rates of the two stages.

4. The entire network is built on a fully connected layer, so why not use the more popular convolutional network?

Response: Thanks for your comments. Convolutional networks are mostly used in the field of image processing, which has two distinctive properties. In natural images, spatially close pixels exhibit stronger and more intricate inter-dependencies. Also, the spatial translation of an object in an image usually does not change its meaning. CNNs have been designed to leverage those two properties. However, neither of these properties hold for scRNA-seq data, for which the ordering of the genes is mostly arbitrary and fixed for all cells. In other words, there is no reason to believe that CNNs are adequate, which is why we uses FC layers. We also explain the reasons for choosing a fully connected layer in the section " Implementation and Parameters Setting " Line 205.

5. In literature, there are already many similar works, e.g. Nucleic Acids Res, 49(D1):D1029-D1037; Bioinformatics. 2021 May 10:btab250, doi: 10.1093/bioinformatics/btab250. The authors should mention related works in their paper.

Response: Thanks for your comments. We have already mentioned these related works in the "Introduction" section and included the corresponding references in the "References" section Line 31 and Line 70.

6. There are some grammatical errors in the manuscript, for example, line 67 “Recently, some new single cell analysis methods based-subspace clustering are proposed”. Please check the entire text carefully and correct any grammatical errors and writing mistakes in the manuscript.

Response: Thanks for your comments. We carefully checked the entire text and corrected grammatical errors and writing mistakes in the manuscript.

**Reviewer 2:**

In this manuscript, the authors proposed a novel model, scDSSC, for clustering single-cell data. This model employs deep sparse subspace clustering by using a self-expressiveness matrix. The extensive experiments show that scDSSC outperforms most competing methods in multiple real datasets.

**Major:**

1. L121. The author should explain why a cell can be represented by other cells in a biological view. This is the assumption of this model.

Response: Thanks for your comments. First, the gene expression profile of a cell can be linearly represented by the gene expression profiles of other cells only within the same subspace. This also means that these cells belong to the same cell type, which refer to their genotypes are the same and the expression patterns of genes within the cells are similar. Therefore, within the same subspace, cells of the same type have strong similarity to each other and can be considered as each other's close neighbors. Representing a cell as a linear combination of near-neighboring cells, which tends to capture global structural information, can yield more reliable similarity. We have also explained this in the " Subspace clustering " section Line 135.

2. Line 63. “However, the cell clusters obtained by these methods lack the support of mathematical theory”. Need more explanations for this statement. The author should give the mathematical theory which only supports the proposed model.

Response: Thanks for your comments. This sentence is a poorly worded sentence that does not convey the meaning what we want. We have made changes to it in the manuscript. What we initially want to express is that scDCC, scDeepCluster and other deep learning methods use deep embedding clustering to generate final clustering results based on a pre-set number of clusters, and the determination of the final cluster number in this process lacks reliable theoretical support, which tends to lead to large deviations between the number of cell clusters and the number of real cell types, affecting the clustering performance. To date, there is no method to accurately estimate the number of cell types in scRNA-seq data. Therefore, the number of clusters is set to the true number of cell types in many models as a way to better evaluate the clustering performance, as is the case in this manuscript. These content also described in the manuscript Line 62.

3. L168. Could the authors explain why they use both MSE and ZINB as the reconstruction loss. Ablation studies are suggested.

Response: Thanks for your comments. The reconstruction loss is only the MSE loss, and we calculate the negative log likelihood of the ZINB distribution as another part of the loss, which is designed for the large number of dropout events in scRNA-seq data. To demonstrate the necessity of simultaneous MSE loss and ZINB loss, several datasets were selected for ablation experiments, and the results are presented in the " Deep sparse subspace clustering " Line 190. The results demonstrate that the model achieves the best clustering performance only when both are in play at the same time.

Table S5 Loss function ablation experiment. Three different conditions were set for the whole experiment, namely, the normal scDSSC model, the loss function without MSE and the loss function without ZINB. The metric NMI was used to assess the clustering performance.

| Datasets | MSE+ZINB | Without ZINB | Without MSE |
| --- | --- | --- | --- |
| 10X_PBMC | 0.7592 | 0.5872 | 0.2983 |
| klein | 0.8831 | 0.6462 | 0.2904 |
| romanov | 0.7068 | 0.5609 | 0.2034 |
| Mouse1 | 0.7881 | 0.5531 | 0.4759 |
| Mouse2 | 0.8092 | 0.5825 | 0.4036 |

4. L259. The author should explain why scDSSC can cope with the batch effect in a computational view.

Response: Thanks for your comments. Different time points, laboratories, platforms or sequencing protocols can introduce some random effects, which introduces batch effect when integrating scRNA-seq data from multiple batches. However, thanks to the self-representation property of subspaces, the gene expression profile of a cell within the same subspace can be represented as a linear combination of the expression profiles of other cells, which tends to capture the global structural information and thus curbs the aforementioned random effects on the integrated data. We have also explained this in the section " Scalability and Robustness for Batch Effect" Line 314.

5. The authors should enhance the resolution of the figures. Many figures are not legible. It is suggested to highlight the differences between scDSSC and scDeepCluster in Figure 1 and 2. From the current figure, I cannot see any differences.

Response: Thanks for your comments. We enhanced the resolution of the images, and we modified Figures 1 and 2 to emphasize their differences from scDCC and scDeepCluster. In addition, we also combined Figure 2 and Figure 1 into one image.

**Minor：**

1. Line 73. “most proposed models subspace-based” should be ‘most proposed subspace-based model’?

Response: Thanks for your comments. We have corrected this error in the manuscript Line 78.

2. Figure 1 and Figure 2 can be combined.

Response: Thanks for your comments. Based on your suggestion, we redrew Figure 1 and merged the original Figure 1 and Figure 2.

3. Line 100. The authors should clarify how to calculate size factor and library size.

Response: Thanks for your comments. They are the same concept, and the size factor can also be called the library size. We also described the calculation process of the size factor in the manuscript from Line 105.

4. Line 100-104. Double-check the grammar.

Response: Thanks for your comments. We double-checked this section and corrected any grammatical errors in it.

5. Figure 2. What are the differences between the red and the orange layers? Please specify.

Response: Thanks for your comments. In the original Figure 2, the red layer is the bottleneck layer of the autoencoder, whose output is a low-dimensional embedding of the original data, and the orange layer is the self-repressive layer we constructed based on the self-representation property, whose output is a matrix of self-repressive coefficients corresponding to the low-dimensional embedding of the original data.

6. It is suggested to put real data description in the method section.

Response: As you suggested, the description of the real data is placed in the "Methods" Line 200.

7. I suggest the authors to include SC3 in the competing methods.

Response: Thanks for your comments. SC3 is a good tool for single-cell data analysis, and at the very beginning, we also intended to compare our approach with SC3. However, after the experiments, we found that SC3 takes more than ten minutes even though the test dataset is a small one containing only thousands of cells, whereas many deep learning models including our method scGMAAE takes only tens of seconds. Furthermore, SC3 also requires a large amount of computational memory, and on our current equipment, we cannot use SC3 to process large-scale single-cell data yet. Therefore, we dropped the comparison with SC3. Here, we chose several small single-cell datasets to test the performance of SC3 and compared the clustering results with our method, as detailed in the table below. The results show that our method outperforms SC3. We also explained this in the manuscript Line 242.

|  | SC3 | | scDSSC | |
| --- | --- | --- | --- | --- |
| **Datasets** | NMI | ARI | NMI | ARI |
| Mouse1 | 0.7192 | 0.4237 | 0.7881 | 0.7258 |
| Mouse2 | 0.6297 | 0.2897 | 0.8092 | 0.8678 |
| 10X_PBMC | 0.7416 | 0.7374 | 0.7592 | 0.7476 |
| Human2 | 0.8150 | 0.6507 | 0.8590 | 0.8855 |
| Romanov | 0.4916 | 0.5182 | 0.7068 | 0.6799 |

8. It is suggested to describe the way of running the competing methods in the method section. For example, for using scDCC, how did the authors build the constraints?

Response: Thanks for your comments. Following your suggestion, we have described the way of running the competing methods in the " Running the competing methods " Line 211.

9. Line 211. ‘We infer that the reason may be that the ZINB distribution cannot well approximate the real distribution of Human kidney dataset.’ The authors may try to run the model without ZINB loss.

Response: Thanks for your comments. According to your suggestion, we do not use ZINB loss to train the model on the Human-kidney dataset, and the results show about the same effect as using ZINB loss. Therefore, we speculate that it may be due to the complex structure of this dataset that it is difficult to learn its optimal multi-subspace representation, which we also explain in the manuscript Line 258.

10. L 283. This is suggested to put this part into the method section.

Response: Thanks for your comments. As you suggested, we have placed the " Implementation and Parameters Setting" in the "Methods" Line 201.

11. I suggest showing NMI in Fig 3 since ACC is influenced by the cluster sizes.

Response: Based on your suggestion, we switch to the NMI metric when presenting clustering performance results, instead of the ACC metric which is easily influenced by cluster size.

12. L226. ‘Next, calculate the nearest neighbors of each cell according to the given number’. The author should explain what this step is for.

Response: Thanks for your comments. This is an operation in scanpy. Before using the UMAP algorithm to reduce the dimension of data, you must first calculate the nearest neighbor graph of each cell. In this process, the number of nearest neighbors as a hyperparameter needs to be specified in advance. We have also explained this in the corresponding place in the manuscript Line 273.

13. In the Umap figures, I cannot find any advantages from scDSSC to the competing methods.

Response: Thanks for your comments. We also used Human1 and Romanov datasets as examples and compared the visualization results with the other seven methods. These can be found in the section "Cell Visualization" of the manuscript Line 281.

14. L272 ‘’ For, example,’ typo

Response: Thanks for your comments. We double-checked the manuscript and corrected similar typos.

**Reviewer 3:**

The study by Zhao et al. proposed a deep sparse subspace clustering method scDSSC. Considering the noise and large number of genes in scRNA-seq data, autoencoder based on ZINB distribution is applied to reduce the dimension and noise of the data simultaneously in scDSSC. The authors showed scDSSC outperformed state-of-the-art methods under various clustering performance metrics.

**Major concern:**

1. Simulated data rather than real data should be used to evaluate the performance of scDSSC, in particular, real data are complexity and could not provide the exactly scenarios under which scDSSC performs better. Furthermore, the clusters in real data are unknown and could not be used to evaluate the performance of these methods. (Fig3–6)

Response: Thanks for your comments. We generated multiple simulation datasets using Splatter, a tool commonly used to generate scRNA-seq simulation data, and they were used to test the performance of scDSSC. The data generation process and all experimental results can be found in the "Simulation Datasets" Line 342.

2. scDSSC clustered the cells into subgroups as previous studies (Fig3–6), which did not provide novel biological insight.

Response: Thanks for your comments. We must admit that this is one of the limitations of our method. The scDSSC mainly starts from the two characteristic properties of high dimensionality and high noise of scRNA-seq data, which may underestimate the distance between cells and make it difficult to identify cell groups. Feature selection, which selects meaningful genes/transcripts from tens of thousands of features, is able to reduce the noise, improve the accuracy of clustering, avoid lost rare cell types and speed up calculations by extracting the informative data and filtering out the disturbing information. However, one major hurdle for the development of feature selection is the various kinds of noise coming from many aspects of scRNA-seq data. One way to solve this problem is to perform subspace clustering to obtain the optimal multi-subspace representation. The shortcoming of this manuscript is that we were not able to apply scDSSC to specific diseases and clinical areas. This is mainly because we have not yet studied specific disease mechanisms, have no researchers in the group who are engaged in clinical research, and have not collected disease-specific single-cell datasets or clinical datasets. Thank you very much for your consideration of our shortcomings, which I believe will be our future endeavor. We also pointed out this limitation in the " Conclusion and Discussion" Line 386.

3. The authors did not provide information on how to establish the reference for judging the performance of these methods based on real data.

Response: Thanks for your comments. All real scRNA-seq datasets are derived from specific literature, and these datasets are considered to be well-annotated. The annotated cell types are treated as real labels, which are not used in the computational process of our model and competing models. Our model outputs the final clustering results, which we treat as predicted labels. Finally, we use the information from the true and predicted labels to measure the performance of the model using three external metrics, ACC, NMI and ARI. We have also added to this in the manuscript Line 240.

**Minor comments:**

1.The manuscript is not well organized and should be carefully organized.

Response: Thanks for your comments. We have reorganized the content and chapters of the manuscript.

2. The fig resolution is low. Some words in fig4 and fig6 could not be recognized.

Response: Thanks for your comments. We redrew the images that appear in the manuscript, improving their resolution.

3. The figure legend is too simple to exactly understand the figure, In particularly Fig4–6.

Response: Thanks for your comments. We added legends to all of the images as a way to convey as much as possible what the images were trying to say, and these are shown in the " Figure Legends" Line 513.

4. Fig4 should be separated into panel A, B, and C for easy reference.

Response: Thanks for your comments. We redrew Figure 4 and divided it into three panels A and B.

5. Fig 5A and 5B are redundant.

Response: Thanks for your comments. Based on your suggestion, we have modified Figure 5, keeping only one of them.

6. Although all the figures legends are appended at the end of the maintext. It is surprised that the fig legend of Fig 3, 4,5 are also in the maintext, which is redundant.

Response: Thanks for your comments. We have removed the legends of Figures 3, 4, and 5 from the maintext.

7. The grammatical errors should be carefully checked.

Response: Thanks for your comments. We carefully checked the full text and corrected the grammatical errors.
