# Supplementary material for: scDSSC: Deep Sparse Subspace Clustering for scRNA-seq Data
Source: PLoS Comput Biol. 2022 Dec 19;18(12):e1010772. doi: 10.1371/journal.pcbi.1010772 (PMC9810169; doi:10.1371/journal.pcbi.1010772)
Supplement: Attachment — Submitted filename: Response letter.docx [file pcbi.1010772.s014.docx]

The Authors’ Response to the Reviewer's Comments on PCOMPBIOL-D-22-01159R1

**“scDSSC: Deep Sparse Subspace Clustering for scRNA-seq Data”**

Hai-Yun Wang, Jian-Ping Zhao, Chun-Hou Zheng and Yan-Sen Su

We sincerely thank the editor and the reviewers for their constructive comments and suggestions concerning our manuscript entitled “scDSSC: Deep Sparse Subspace Clustering for scRNA-seq Data” (ID: PCOMPBIOL-D-22-01159R1). We have carefully revised the manuscript according to these constructive comments. The responds to the reviewer’s comments are listed as follows.

**Reviewer reports:**

**Reviewer 2:**

The authors addressed most of my concerns. Some claims should be further clarified.

1. The authors state that “the gene expression profile of a cell can be linearly represented by

the gene expression profiles of other cells only within the same subspace. This also means that

these cells belong to the same cell type, which refers to their genotypes being the same and the

expression patterns of genes within the cells are similar.” This statement is misleading because

the cell-type information (ground truth) cannot be used for clustering. Do the authors mean that

a cell is represented by the cells that are predicted to be in the same cell type? Besides, how do

the authors initialize C? This step might be very important for this model.

Response: Thank you very much for your comments. We have carefully discussed and considered your comments, and our previous statements were indeed somewhat misleading. As you said, our assumption is that the expression profile of a cell can be represented by the expression profiles of other cells predicted to be of the same type. We have modified the previous statement in the manuscript. The matrix C is a coefficient matrix, also called a self-expressive matrix (SEM), which is a square matrix whose dimension is equal to the number of cells. In this work, we initialize C using the identity matrix and then continuously update C based on the loss function of the model and the regularized loss imposed on C.

2. The authors state that “However, thanks to the self-representation property of subspaces,

the gene expression profile of a cell within the same subspace can be represented as a linear

combination of the expression profiles of other cells, which tends to capture the global structural

information and thus curbs the aforementioned random effects on the integrated data.” I believe

this is only working when the batch effects are smaller than the differences between different

cell types. The authors should specify this in the manuscript.

Response: Thanks for your comments. We have specified this in the corresponding place in the manuscript.

3. The authors state that “When using scDCC, the “constraint-pairs” are first computed

according to the way proposed in the paper, and then a semi-supervised learning is performed”.

The performance of scDCC is majorly depending on the constraints used. It is suggested to

specify the way of building constraints, such as the marker gene(s) used as well as the number

of ML and CL links.

Response: Thanks for your comments. Unlike traditional hard-constrained clustering algorithms, in scDCC, the authors convert (partial) prior knowledge into soft pairwise constraints and add them as additional terms into the loss function for optimization. The pairwise constraint can have two types: must-link (ML) and cannot-link (CL). I have also explained this in the manuscript.

4. If SC3 takes too large RAM or too long times, it is suggested using a subset of cells to run

the experiments.

Response: Thanks for your comments. We tested the performance of SC3 with all 14 datasets by borrowing virtual memory, including larger datasets with more than 10,000 cells. We evaluated the clustering results of SC3 on each dataset using both NMI and ARI metrics, and compared SC3 with our method, and the specific results are listed in the following table. The results in the table show that SC3 outperforms our method scDSSC in clustering on two datasets, Zeisel and Klein, but scDSSC achieves better results on all the remaining 12 datasets. We also mention these experimental results in the manuscript Line 248.

|  | SC3 | | scDSSC | |
| --- | --- | --- | --- | --- |
| **Datasets** | NMI | ARI | NMI | ARI |
| Mouse1 | 0.7192 | 0.4237 | 0.7881 | 0.7258 |
| Mouse2 | 0.6297 | 0.2897 | 0.8092 | 0.8678 |
| 10X_PBMC | 0.7416 | 0.7374 | 0.7592 | 0.7476 |
| Human2 | 0.8150 | 0.6507 | 0.8590 | 0.8855 |
| Romanov | 0.4916 | 0.5182 | 0.7068 | 0.6799 |
| Human1 | 0.7800 | 0.5661 | 0.8707 | 0.8086 |
| Human3 | 0.8265 | 0.7204 | 0.8009 | 0.8082 |
| Human4 | 0.8021 | 0.5964 | 0.8268 | 0.8335 |
| Zeisel | 0.7870 | 0.8497 | 0.6340 | 0.6391 |
| Klein | 0.9980 | 0.9993 | 0.8831 | 0.9161 |
| CITE_CBMC | 0.4183 | 0.1320 | 0.6697 | 0.5816 |
| Human_kidney | 0.5964 | 0.4717 | 0.6582 | 0.5152 |
| HumanLiver | 0.5192 | 0.2550 | 0.8130 | 0.8447 |
| Macosko_mouse | 0.3281 | 0.0228 | 0.8161 | 0.8010 |

5. Some texts on the figures are still too small to read.

Response: Thanks for your comments. We have modified some of the images to enlarge the font in them.
